# Affinity Captured Urinary Extracellular Vesicles Provide mRNA and miRNA Biomarkers for Improved Accuracy of Prostate Cancer Detection: A Pilot Study

**DOI:** 10.3390/ijms21218330

**Published:** 2020-11-06

**Authors:** Michelle Davey, Sami Benzina, Marc Savoie, Guy Breault, Anirban Ghosh, Rodney J. Ouellette

**Affiliations:** 1 Atlantic Cancer Research Institute, Moncton, NB E1C 8X3, Canada; MichelleD@canceratl.ca (M.D.); sami.benzina@umontreal.ca (S.B.); anirgho@gmail.com (A.G.); 2 Dr. Georges-L.-Dumont University Hospital Centre, Moncton, NB E1C 2Z3, Canada; Marc.Savoie@vitalitenb.ca (M.S.); Guy.Breault@vitalitenb.ca (G.B.)

**Keywords:** prostate cancer, extracellular vesicles, urine, mRNA, miRNA, biomarkers, diagnostic

## Abstract

Serum prostate-specific antigen (sPSA) testing has helped to increase early detection of and decrease mortality from prostate cancer. However, since sPSA lacks specificity, an invasive prostate tissue biopsy is required to confirm cancer diagnosis. Using urinary extracellular vesicles (EVs) as a minimally invasive biomarker source, our goal was to develop a biomarker panel able to distinguish prostate cancer from benign conditions with high accuracy. We enrolled 56 patients in our study, 28 negative and 28 positive for cancer based on tissue biopsy results. Using our Vn96 peptide affinity method, we isolated EVs from post-digital rectal exam urines and used quantitative polymerase chain reaction to measure several mRNA and miRNA targets. We identified a panel of seven mRNA biomarkers whose expression ratio discriminated non-cancer from cancer with an area under the curve (AUC) of 0.825, sensitivity of 75% and specificity of 84%. We also identified two miRNAs whose combined score yielded an AUC of 0.744. A model pairing the seven mRNA and two miRNA panels yielded an AUC of 0.843, sensitivity of 79% and specificity of 89%. Addition of EV-derived PCA3 levels and clinical characteristics to the biomarker model further improved test accuracy. An AUC of 0.955, sensitivity of 86% and specificity of 93% were obtained. Hence, Vn96-isolated urinary EVs are a clinically applicable and minimally invasive source of mRNA and miRNA biomarkers with potential to improve on the accuracy of prostate cancer screening and diagnosis.

## 1. Introduction

Worldwide, prostate cancer is the second most frequent cancer diagnosis and the fifth leading cause of cancer death in men [1]. Although introduction of the serum prostate-specific antigen (sPSA) test has helped to increase early detection and decrease mortality from prostate cancer, the test has limitations that have made its use in screening highly controversial [2,3,4,5,6,7]. First, sPSA is not specific for cancer; other conditions such as inflammation, trauma, benign prostate hyperplasia (BPH), and prostatic intraepithelial neoplasia (PIN) can also cause sPSA elevation. Secondly, sPSA cannot distinguish indolent, low-risk prostate cancer from aggressive, high-risk tumours. Between 20–50% of cancers detected through sPSA screening are overdiagnosed [3], meaning that if never detected they would not likely have caused symptoms or death. Hence, a large percentage of men either without cancer or with clinically insignificant prostate cancer are unnecessarily exposed to the risks of invasive prostate biopsies for definitive diagnosis [8]. Conversely, a low sPSA level does not guarantee the absence of prostate cancer. A false negative rate of about 15% is associated with the test [9]. Active surveillance and watchful waiting have emerged as strategies to reduce the economic and physical burdens associated with overtreatment of low-risk tumours. However, neither program eliminates the issue of overdiagnosis, which in itself is associated with reduced quality of life [2,10]. Moreover, active surveillance requires the patient to undergo routine sPSA tests, digital rectal exams (DREs), repeat biopsies, and imaging (e.g., TRUS, MRI) to track the cancer’s status; these procedures have potentially serious risks and psychological harms [11,12,13]. The limitations of sPSA screening and prostate tissue biopsies highlight the urgent need for minimally invasive biomarkers with improved specificity and sensitivity for prostate cancer detection, diagnosis and monitoring.

Liquid biopsy, the analysis of biomarkers from body fluids such as blood and urine, has emerged as an innovative minimally invasive tool for disease detection and monitoring [14,15]. Liquid biopsies are associated with significantly less morbidity and can be scheduled more frequently than traditional tissue biopsies. Moreover, biomarkers interrogated from liquid biopsies have a better chance of reflecting the heterogeneous nature of prostate cancer and hence can provide a more complete molecular profile of disease status at any given time [14,15,16]. Urine, in particular, is a promising fluid to investigate for diagnostic prostate cancer biomarkers, and several tests have been developed, but these tests lack the combination of high sensitivity and specificity required for use in accurate detection and diagnosis of prostate cancer [17,18,19,20,21,22,23,24,25,26,27].

Isolation of extracellular vesicles (EVs) from urine is an emerging approach to improve on the sensitivity and specificity of biomarkers for prostate cancer detection and diagnosis. EVs are nano-sized (30–1000 nm) membrane bound structures that are either passively released or actively secreted by cells and can be found in several body fluids, including urine [28]. They contain a protected and selectively enriched repertoire of proteins, nucleic acids and metabolites derived from their originating cells. Moreover, cancer cells are known to produce an overabundance of EVs whose functional roles in intercellular communication, tumour transformation, invasiveness, and growth are coming to light [15,29,30,31].

We have developed a clinically-applicable EV isolation method that uses a synthetic peptide called Vn96. The peptide has binding affinity for heat shock proteins that are abundantly expressed on the surface of EVs [32]. EVs bound with the peptide quickly form aggregates that are readily separated from their liquid milieu by low-speed centrifugation. We and others have previously demonstrated the validity of our EV capture method for cell culture supernatants, blood and urine [32,33,34,35,36].

In a previous study we analyzed tissue microarray data sets to identify a panel of eight messenger RNAs (mRNAs) useful in prostate cancer diagnosis [37]. The reference-free diagnostic panel used the ratio of relative expression of three mRNAs that are overexpressed (*FOLH1*, *XBP1* and *HPN*) to five mRNAs that are underexpressed (*ITSN1*, *GSTM4*, *LTBP4*, *NELL2*, and *CFD*) in prostate cancer compared to normal tissue. Using RNA isolated from prostate tissues to validate the panel, an area under the curve (AUC) of 0.955 with specificity and sensitivity of 90% was achieved for discrimination of cancer from normal.

In this study we sought to demonstrate the value of Vn96 peptide-mediated isolation of urinary EVs as a source of both prostate-specific and clinically-relevant RNA biomarkers for prostate cancer detection and diagnosis. Additionally, we assessed whether the discriminatory power of the eight mRNA reference-free panel, as previously determined using prostate tissue samples [37], is translatable to a non-invasive urine sample source, with a specific focus on EV RNA.

## 2. Results

### 2.1. Derivation of a Vn96-Isolated EV Reference-Free mRNA Panel for Prostate Cancer Diagnosis

**Figure 1:**
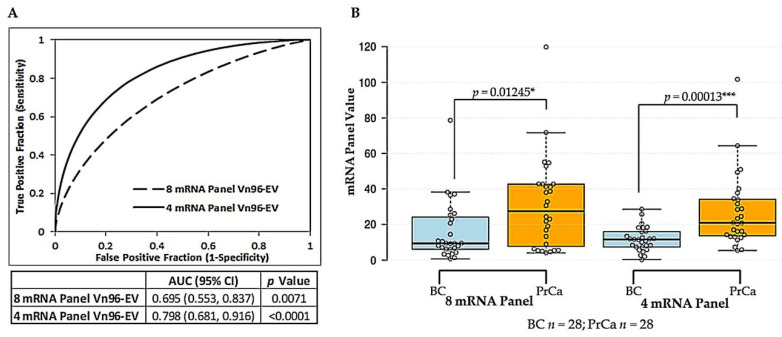
Comparison of Vn96-isolated EV reference-free eight mRNA versus four mRNA panels for prostate cancer discrimination. (**A**) ROC curve analysis showing discrimination of Prostate Cancer (PrCa; *n* = 28) from Benign Control (BC; *n* = 28) using either the eight mRNA or optimized four mRNA reference-free panel values obtained with Vn96-isolated EV RNA. AUC, 95% CI and *p* value are provided below the plot. (**B**) Box plot graphically depicting results of eight mRNA and four mRNA panels obtained for Benign Control (BC) versus Prostate Cancer (PrCa) groups using Vn96-isolated EV RNA. The central boxes represent the values from the lower to upper quartiles (25th to 75th percentiles). Lines within the boxes are the median values (50th percentile). Whiskers extending from the boxes indicate the minimum to maximum values obtained (excluding outliers, which are displayed as separate points). *p* values were obtained using the Mann-Whitney U test to compare differences. Differences denoted by asterisks are statistically significant (* *p* < 0.05 and *** *p* < 0.001). Values for the eight mRNA panel were calculated as the ratio of expression of three mRNAs overexpressed in prostate cancer (*FOLH1*, *XBP1* and *HPN*) to five mRNAs underexpressed in prostate cancer (*ITSN1*, *GSTM4*, *LTBP4*, *NELL2*, and *CFD*). The optimized four mRNA panel used the ratio of expression of *FOLH1* and *HPN* (over) to *GSTM4* and *CFD* (under).

Our previously identified eight-member mRNA panel for prostate cancer (based on prostate tissue RNA results) [37] yielded an AUC of 0.695 with a 95% confidence interval (CI) of 0.553–0.837 when measured using Vn96-isolated EV RNA from a set of 28 prostate cancer and 28 benign control post-digital rectal exam (DRE) urine samples. Characteristics of the patients enrolled in the study are provided in Table 1. However, two of the mRNAs, *LTBP4* and *NELL2*, could not be measured in several prostate cancer and benign control samples. Removal of these genes from the panel had little impact on AUC values. Logistic regression analysis was used to assess the influence of removal of other mRNAs from the panel using one-by-one elimination. Results for Vn96-isolated EVs are shown in Table 2. Leaving *XBP1* out of the calculation (in addition to *LTBP4* and *NELL2*) resulted in the largest improvement in predictive power with a resultant *p* value of 0.003, compared to 0.02 for the original eight mRNA panel. Removal of *GSTM4* also improved predictive significance (*p* value of 0.012). The resulting four mRNA reference-free panel, consisting of *FOLH1*, *HPN* and *ITSN1* (overexpressed genes), and *CFD* (underexpressed genes), yielded an odds ratio (OR) of 1.136 with a 95% CI of 1.0479–1.2313. The associated *p* value was 0.002. Receiver operating characteristic (ROC) curve analysis showed an improvement in diagnostic accuracy from AUC = 0.695 for the original eight mRNA panel to AUC = 0.798 (95% CI = 0.681–0.916) for the resulting four mRNA panel (Figure 1A). Application of the Mann-Whitney U test confirmed the difference between benign control and cancer groups was dramatically more significant using the four mRNA panel (*p* value = 0.00013) versus the eight mRNA panel (*p* value = 0.0125) (Figure 1B). We performed the same analyses for sucrose cushion ultracentrifugation (scUCF)-isolated EV RNA and sediment RNA (Supplemental Table S1). The results were similar to Vn96-isolated EV RNA, in that removal of *LTPB4*, *NELL2*, *XPB1,* and *GSTM4* improved AUC values from 0.505 to 0.64 for scUCF-isolated EV RNA, and from 0.614 to 0.676 for sediment RNA.

**Figure 2:**
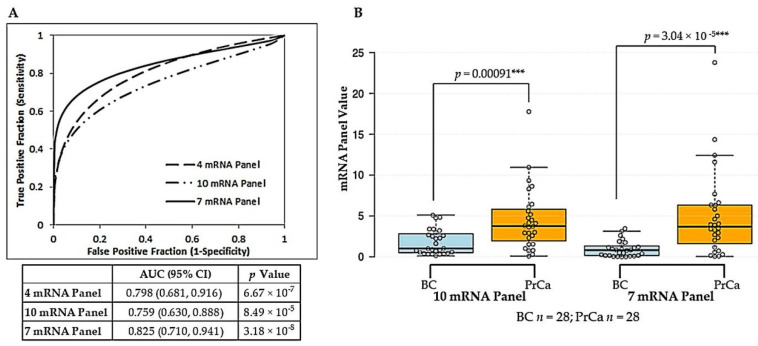
Comparison of Vn96-isolated EV reference-free mRNA panels for prostate cancer discrimination: derivation of seven mRNA panel. (**A**) ROC curve analysis showing discrimination of Prostate Cancer (PrCa; *n* = 28) from Benign Control (BC; *n* = 28) using either the previously optimized four mRNA panel, the 10 mRNAs (four mRNAs from panel plus six mRNAs sourced from literature) as a combined panel, or the final optimized seven-member mRNA panel. AUC, 95% CI and *p* value for each reference-free mRNA panel are provided below the plot. (**B**) Box plot graphically depicting results for the 10 mRNA and the optimized seven mRNA panels obtained for Benign Control (BC) versus Prostate Cancer (PrCa) groups using Vn96-isolated EV RNA. Details of plot are as described for Figure 1. *p* values were obtained using the Mann-Whitney U test to compare differences. Differences denoted by asterisks are statistically significant (*** *p* < 0.001). The final seven mRNA panel was calculated as the ratio of expression of four mRNAs overexpressed in prostate cancer (*FOLH1*, *HPN, CD24, TMPRSS2-ERG*) to three mRNAs underexpressed in prostate cancer (*ITSN1, ANXA3, SLC45A3*).

Although promising, these results did not mirror the high diagnostic power we had obtained with the original eight mRNA panel for prostate tissues. We therefore assessed whether the addition of other mRNAs reported in the literature to be dysregulated in prostate cancer could improve performance of the reference-free model in the liquid biopsy setting. *GOLM1*, *CD24*, *PSCA,* and the gene fusion *TMPRSS2-ERG* were selected as overexpressed mRNAs in prostate cancer, whereas *ANXA3* and *SLC45A3* were selected as underexpressed mRNAs in prostate cancer for evaluation. The resultant 10 mRNA reference-free panel yielded an AUC of 0.759 (95% CI = 0.630–0.888). Logistic regression analysis was used to assess the contribution of each mRNA to the predictive power of the panel (Table 3). The combined removal of *GOLM1*, *PSCA* and *CFD* led to the derivation of a seven-member mRNA panel (*FOLH1*, *HPN*, *CD24*, *TMPRSS2-ERG* overexpressed; *ITSN1*, *ANXA3*, *SLC45A3* underexpressed) with increased significance for prediction of prostate cancer over the 10-member panel (seven mRNA panel: OR = 2.237, 95% CI = 1.4036–3.5656, *p* value = 0.0007; 10 mRNA panel: OR = 1.636, 95% CI = 1.1808–2.2663, *p* value = 0.0031) and the previously derived four mRNA panel (OR = 1.136, 95% CI = 1.0479–1.2313, *p* value = 0.002). The improved diagnostic accuracy of the seven mRNA panel was confirmed by ROC curve analysis, which showed an increase in AUC to 0.825 (95% CI = 0.710–0.941) (Figure 2A). At the optimal cut-off threshold, a sensitivity of 75% (95% CI = 55–89%) and a specificity of 84% (95% CI = 67–96%) was achieved. The associated Positive Predictive Value (PPV) was 84% (95% CI = 64–94%) with a Negative Predictive Value (NPV) of 77% (95% CI = 58–93%). The difference between benign control and prostate cancer sample groups was highly significant using the seven mRNA panel (Figure 2B).

### 2.2. Derivation of a Vn96-Isolated EV Combined mRNA and miRNA Model for Prostate Cancer Diagnosis

**Figure 3:**
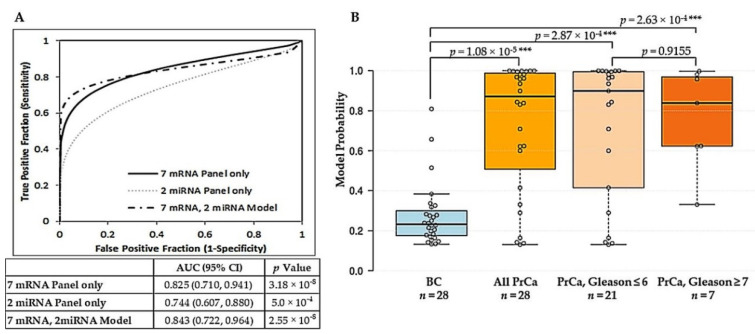
Combined urinary Vn96-isolated EV seven mRNA and two miRNA model for prostate cancer discrimination. (**A**) ROC curve analysis showing discrimination of Prostate Cancer (PrCa; *n* = 28) from Benign Control (BC; *n* = 28) using the optimized seven mRNA reference-free panel, the two miRNA panel result (geometric mean of miR-375-3p and miR-574-3p), and the model combining the panels. AUC, 95% CI and *p* value for each panel and the model are provided below the plot. (**B**) Box plot graphically depicting prostate cancer classification probabilities calculated using the seven mRNA/2 miRNA model for Benign Controls (BC; *n* = 28), all Prostate Pancer (PrCa; *n* = 28), PrCa Gleason 6 or below (*n* = 21), and PrCa Gleason 7 or above (*n* = 7). Plot details are as for Figure 1. Differences denoted by asterisks are statistically significant (*** *p* < 0.001). *p* values were obtained using the Mann-Whitney U test to compare differences.

The altered expression of several microRNAs (miRNAs) has been shown in tissue, blood and urine samples of prostate cancer patients [38,39,40,41]. Candidate miRNA biomarkers, selected from the literature, were screened for their presence in urinary Vn96-isolated EVs using a small set of samples, and miR-141-3p, miR-375-3p, miR-574-3p, and miR-21-3p were selected for further evaluation (a list of screened miRNAs is provided in Supplemental Table S2). Univariate logistic regression showed that each miRNA, individually, had significant power to predict prostate cancer (Table 4) with *p* values all <0.05 and ranging from 0.0211 for miR-574-3p to 0.0357 for miR-21-3p. Combination of the miRNAs further improved their predictive power. Logistic regression and ROC curve analysis showed that the combination of miR-375-3p and miR-574-3p yielded the best diagnostic ability (AUC = 0.744). We then wished to assess whether this two miRNA panel could add value to the optimized seven mRNA reference-free panel. Multivariable logistic regression was done to assess performance of the model (Table 4). Within this model, the seven mRNA panel was found to be an independently significant predictor, with a more robust OR (OR = 1.8463, 95% CI = 1.1375–2.9968) than the two miRNA panel (OR = 1.1822, 95% CI = 0.9581–1.4587), which was not independently significant. On ROC curve analysis, the model combining the seven mRNA panel with the two miRNA panel resulted in an AUC = 0.843 (95% CI = 0.722–0.964) with sensitivity and specificity of 79% (95% CI = 59–92%) and 89% (95% CI = 72–98%), respectively (Figure 3A). A PPV of 88% (95% CI = 69–96%) with corresponding NPV of 81% (95% CI = 62–96%) was obtained. The difference between model probabilities for the benign control group versus the prostate cancer group was highly significant (*p* value = 1.08 × 10^−5^) (Figure 3B). At the optimal cut-off of the model, 76% of samples with assigned biopsy Gleason scores of 6 or less and 86% of samples with assigned Gleason scores of 7 or above were correctly assigned as prostate cancer; however, the difference in probabilities between the Gleason groups was not significant for their discrimination (Figure 3B).

### 2.3. Additive Value of Combining Vn96-Isolated EV mRNA and miRNA Panels with PCA3 and Clinical Characteristics

**Figure 4:**
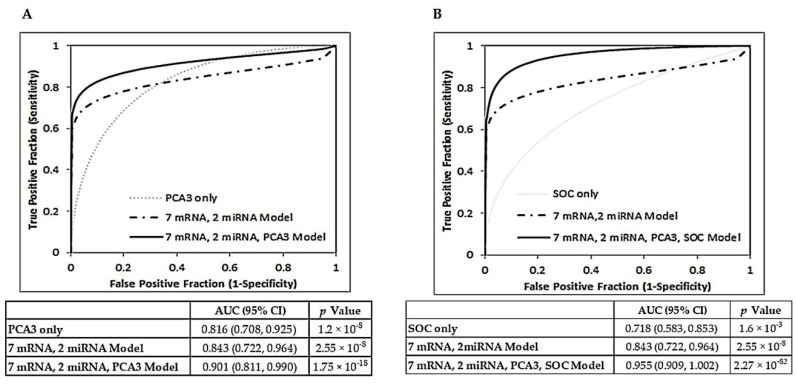
Multivariable models combining urinary Vn96-isolated EV 7 mRNA/2 miRNA panels with Vn96-isolated EV PCA3 values and clinical characteristics. (**A**) ROC curve analysis showing discrimination of Prostate Cancer (PrCa; *n* = 28) from Benign Control (BC; *n* = 28) using Vn96-isolated EV PCA3 values only, the seven mRNA/2 miRNA model only, and a multivariable model incorporating PCA3 with the sevn mRNA/2 miRNA values. (**B**) ROC curve analysis showing discrimination of Prostate Cancer (PrCa; *n* = 28) from Benign Control (BC; *n* = 28) using a model combining clinical characteristics observed as part of prostate cancer standard of care (SOC) only, the seven mRNA/2 miRNA model only, and a multivariable model including Vn96-isolated EV PCA3 results with seven mRNA/2 miRNA values and SOC characteristics. AUC, 95% CI and *p* values are provided below the plots.

Univariate logistic regression revealed that, independently, Vn96-isolated EV *PCA3* value (Supplemental Figure S2) was a modest but significant predictor of prostate cancer classification (OR = 1.0960, 95% CI = 1.0298–1.1665, *p* value = 0.0039) (Table 5). Conversely, the clinical characteristics of age, sPSA level, prostate volume, and DRE result were neither strong nor independently significant predictors of diagnosis. In a multivariable model combining these clinical characteristics, ROC curve analysis demonstrated modest diagnostic ability with an AUC = 0.718 (Model 1, Table 5). A model combining the Vn96-isolated EV seven mRNA reference-free panel, two miRNA panel and *PCA3* values yielded an AUC of 0.901, which was an improvement in diagnostic accuracy over the seven mRNA/2 miRNA model (AUC = 0.843) and *PCA3* alone (AUC = 0.816) (Model 2, Table 5, Figure 4A). Within this model, the seven mRNA panel remained a strong, independent predictor. Contribution of *PCA3* value to the model was modest but independently significant. Model 3 added clinical characteristics to Model 2 (Table 5). The Vn96-isolated EV seven mRNA panel and two miRNA panel remained strong contributors to the predictive ability of the model, with ORs of 1.4312 and 1.4024, respectively. sPSA also proved to be a good contributing predictor within Model 3 (OR = 1.522). However, the predictive powers of the RNA panels and sPSA were each dependent on other model variables (*p* values > 0.05). ROC curve analysis revealed Model 3 to have excellent accuracy for prostate cancer discrimination from benign controls with an AUC of 0.955 (95% CI = 0.909–1.002) (Figure 4B). At the optimal cut-off point, sensitivity was 86% (95% CI = 67–96%) with an associated specificity of 93% (95% CI = 77–91%). The PPV of the model was 92% (95% CI = 75–98%) with a NPV of 87% (95% CI = 69–98%).

## 3. Discussion

Prostate cancer detection is currently based on sPSA, DRE and, when available, multiparametric magnetic resonance imaging (mpMRI) results [2]. The diagnosis is confirmed by pathological examination of biopsied prostate tissue [3,5]. Much of the controversy surrounding the use of sPSA is based on its low specificity for cancer [8]. A second issue related to the use of sPSA screening is overdiagnosis and overtreatment of indolent disease, since sPSA cannot distinguish between slow-growing and aggressive prostate cancer. Although active surveillance has emerged as a strategy to limit overtreatment, patients are still required to undergo routine testing (sPSA, DRE, imaging) and repeat biopsies to monitor the cancer [2]. Hence, there is a need for development of non-invasive biomarkers to improve the accuracy of prostate cancer early detection, diagnosis and management.

We previously identified a panel of eight mRNAs with potential for the detection and diagnosis of prostate cancer [37]. The reference-free mRNA panel was able to discriminate cancer from non-cancer with high accuracy (AUC = 0.955) using biopsied prostate tissues. In the current study, we examined the diagnostic potential of this mRNA panel using urine as a non-invasive ‘liquid biopsy’ sample. We also demonstrated the utility of our Vn96 peptide-mediated EV capture technology as a clinically feasible liquid biopsy tool that yields diagnostically-relevant mRNA and miRNA biomarkers for prostate cancer.

Liquid biopsy, the examination of tumour-derived material in body fluids (such as blood, saliva, cerebrospinal fluid and urine), has gained momentum as a non- or minimally-invasive means to assess the presence and status of cancer [18,30,42,43]. In the field of urological cancers, urine is a promising biofluid to examine for biomarkers [44,45]. EVs have several properties that make them attractive targets for biomarker discovery [29,30,46]. EVs contain a repertoire of molecules (DNAs, RNAs, proteins, and lipids) derived from the cell of origin. These contents are stable, as they are protected from degradation within the lipid bilayer of the EV [47,48]. EVs are abundant in body fluids and are actively released in large numbers by tumour cells. Moreover, there is increasing evidence that EV cargo is not randomly packaged, but rather selectively sorted depending on the intended functional role of the EV in tumour transformation, invasiveness and growth [49,50,51,52,53,54]. Although recognized as rich biomarker sources, a major hindrance to the application of EVs in diagnostics has been the limited availability of clinically-amenable methods for their isolation from biofluids. The Vn96 peptide represents a unique clinically amenable method for EV isolation that is simple, quick, and efficient and does not require specialized laboratory equipment [32,33].

As part of this study, we verified that urinary EVs captured by our simple Vn96 peptide method are comparable to those isolated using a commonly employed ultracentrifugation method (Supplemental Figure S1). We demonstrated the presence of canonical EV markers such as CD9, CD63 and PDCD6IP, as well as prostate-specific markers (FOLH1 and KLK3) in Vn96-isolated EV samples. The results highlight the efficacy of Vn96 peptide for EV capture, as in most cases EV-specific proteins were highly enriched in Vn96-isolated EV preparations. Although the most common method for EV isolation, ultracentrifugation is a time-consuming multi-step process that can lose as much as 40–60% of vesicles present in the sample [55,56]. Post-DRE samples showed higher levels of prostate-specific proteins and EV markers compared to pre-DRE samples (Supplemental Figure S1). Increased protein and EV levels in urine following DRE have been previously observed [45,57,58] and are not surprising considering the anatomical location of the prostate gland. Thus, to optimize the chance of successfully applying our reference-free mRNA panel to prostate cancer diagnosis in urine we continued our study using post-DRE samples.

As a benchmark to demonstrate the presence of diagnostically-relevant prostate-related RNAs in Vn96-isolated urinary EVs, we assessed *PCA3* to *KLK3* ratios using this material. RNAs from urine sediments and scUCF-isolated EVs were also examined (Supplemental Figure S2). *PCA3* is a long non-coding RNA originally detected in urine sediments following prostatic massage and is a recognized upregulated marker of prostate cancer [23,59,60,61]. Both *PCA3* and *KLK3* were readily measured using RNA from Vn96-isolated EVs, and results were comparable to those of scUCF-isolated EVs (Supplemental Figure S2). The results suggested that EVs captured using our quick and simple Vn96 method carried clinically relevant transcripts in abundance ratios similar to EVs isolated using the tedious, time-consuming and clinically unfeasible scUCF method.

Despite successfully demonstrating that post-DRE urine contains EVs with prostate-related proteins and mRNAs, the high discriminatory power of our eight mRNA reference-free panel for prostate cancer, as determined using prostate tissues, was not translatable to urine samples. The AUC was only 0.695 using urinary EVs as the RNA source (Table 2). Via logistic regression analysis we eliminated mRNAs from the panel that did not contribute to predictive ability; however, the resulting four mRNA reference-free panel still had only moderate diagnostic accuracy for prostate cancer with an AUC of 0.798 usingVn96-isolated EV RNA (Figure 1). EV cargos have long been thought to mirror the contents of their parental cells; however, recent research suggests that packaging and sorting of nucleic acids and proteins into EVs is not random and that the RNA content of EVs can be markedly different from that of the originating cell. A popular theory is that parent cells selectively package molecules into EVs based on what is required to exert their desired regulatory effects on the recipient cells [49,51,62]. These observations may explain why our tissue-derived eight mRNA panel was not translatable to urinary EVs, which may only carry a selection of RNA cargo from the parent cells.

To boost confidence in the diagnostic utility of urinary EVs captured using theVn96 peptide, we performed a literature search for prostate cancer-associated mRNA targets previously detected in liquid biopsies. We were able to measure the expression of several putative prostate cancer biomarkers using urinary Vn96-isolated EV material. Through logistic regression and ROC curve analysis we derived a seven mRNA reference-free biomarker panel (combining mRNAs from our original eight-member panel with those from literature) that has good accuracy (AUC = 0.825) for discriminating cancer from benign controls (Figure 2 and Table 3). The panel consists of *FOLH1* [63,64], *HPN* [64,65], *CD24* [66,67], and *TMPRSS2-ERG* [68,69] as overexpressed mRNAs with *ITSN1* [37,70], and *ANXA3* [71,72] and *SLC45A3* [73,74] as underexpressed mRNAs. Although these biomarkers have been studied for prostate cancer diagnostics previously, their measurement in Vn96-captured urinary EVs and combination into a mRNA panel for the discrimination of prostate cancer from benign conditions is novel. The use of a mRNA expression ratio (mean of overexpressed mRNAs divided by mean of underexpressed mRNAs) eliminates the need for correction to an endogenous reference mRNA(s), which is of particular relevance in the liquid biopsy field. Given the complexity of EV populations within a biofluid sample and the selective sorting of RNA cargo into EVs, reference mRNAs that are often used for normalization of cellular RNA samples are not necessarily suitable for normalizing EV RNA data. Selection of appropriate normalizers for EV RNA can be a challenging process requiring extensive evaluation [75,76,77].

miRNAs have emerged as key components of EV cargo. These small (20–25 nucleotides) molecules have distinct roles in gene regulation and many have been implicated in cancer pathogenesis [29,39,41]. For prostate cancer, the majority of studies exploring circulating miRNAs have done so using plasma or serum. Very few studies have examined miRNA expression levels in urine. Of the many putative prostate cancer miRNA markers, miR-141, miR-375, miR-574, and miR-21 are among those most studied with expression levels correlated to cancer stage, Gleason score and metastasis [77,78,79,80,81,82,83,84,85]. Our examination of these miRNAs in Vn96-isolated EVs from urine samples showed similar results. Individually, the selected miRNAs (miR-141-3p, miR-375-3p, miR-574-3p, and miR-21-3p) had significant power to discriminate prostate cancer from benign control groups. A combination of miR-375 and miR-574 yielded the best diagnostic ability (Table 4). Integration of this Vn96-isolated EV 2 miRNA panel with the Vn96-isolated EV seven mRNA panel resulted in a multivariable model able to significantly distinguish cancer from benign with an AUC of 0.843 (PPV = 88%, NPV = 81%) and associated sensitivity and specificity values of 79% and 89%, respectively (Figure 3). Given that patients in the biopsy negative (benign control) group presented with other prostate conditions, such as BPH and PIN and/or abnormal DRE (Table 1), the results bode well for a clinically-applicable diagnostic test able to distinguish non-cancerous prostate conditions from prostate cancer.

Although several urine-based RNA biomarker tests have been developed for prostate cancer diagnosis, they have limited accuracy. For example, *PCA3* has an AUC of 0.7–0.8 with sensitivity around 64% and specificity around 76% [23,24]. Of note, the addition of Vn96-isolated EV *PCA3* values to our seven mRNA/2 miRNA model improved the accuracy of prostate cancer diagnosis to an AUC of 0.9 (Figure 4). These results highlight the improved potential for multi-marker panels, as opposed to any one single biomarker, to more accurately reflect the highly heterogenous nature of prostate cancer. Another marker, *TMPRSS-ERG*, by itself has a sensitivity of about 24%, specificity of about 93%, and an AUC of 0.84 [86]. The combination of *TMPRSS-ERG* with *PCA3* has good sensitivity (around 88%) but lacks specificity (around 49%) [27]. Measurement of *PCA3*, *GOLPH2*, *SPINK1,* and *TMPRSS2: ERG* in a multi-gene assay had an AUC of 0.76 with sensitivity of 67% and specificity of 76% [22]. In comparison, our model combining mRNA and miRNA panels measured using RNA from urinary Vn96-isolated EVs, has a better combination of sensitivity (79%), specificity (89%) and AUC (0.84) values that may translate to a more accurate non-invasive diagnostic test for prostate cancer.

For the patient group in this study, combining standard prostate cancer clinical characteristics (age, serum PSA level, prostate volume and DRE results) into a multivariable model yielded modest diagnostic accuracy (AUC = 0.718). However, the addition of our Vn96-isolated EV seven mRNA panel and two miRNA panel to the model improved the AUC to 0.929. Inclusion of Vn96-isolated EV *PCA3* values within the model further improved predictive power with a resultant AUC of 0.955, sensitivity of 86%, and specificity of 93%. Hence, in conjunction with clinical characteristics, our urinary Vn96-isolated EV RNA panels could improve accuracy of prostate cancer detection and hence reduce the number of unnecessary biopsies.

We recognize that there are several limitations to the current study. The sample size is modest; however, the main purpose of the study was to demonstrate that our quick and simple Vn96 peptide method can isolate EVs from urine enriched with clinically relevant prostate cancer RNA biomarkers. Further investigation with a much larger sample number is needed to validate our findings. Moreover, the standard we used to confirm cancer diagnosis was TRUS-guided biopsy results, a method with an associated false-negative rate ranging from 15–46% and a rate of about 38% for under-grading prostate cancer when compared to Gleason score on radical prostatectomy [87]. Additionally, after assessing expression levels of various small nucleolar RNAs in our Vn96-isolated EV RNA samples, we chose to use *SNORD44* for normalization of miRNA results. As is the case for mRNAs, there is currently no consensus on endogenous reference controls for EV miRNAs. Hence, although our miRNA results were in concordance with the literature, our choice of *SNORD44* for normalization of miRNA levels may not have properly accounted for expression differences across patient samples. Lastly, since the DRE procedure may be uncomfortable and is associated with a degree of non-compliance, it would be of great interest to assess the diagnostic potential of Vn96-isolated EVs for prostate cancer using first morning void or mid-stream urine.

## 4. Materials and Methods

### 4.1. Study Participants

Under Institutional Review Board approval (Vitalité Health Network Research Ethics Board; CER-2011-08; approved March 2011 and renewed yearly) and with patients’ informed consent, we collected freshly voided urine samples from a total of 56 males at the Urology Clinic of the Georges-L.-Dumont University Hospital Centre (Moncton, NB, Canada). All patients participated in a consult for a prostate problem or an elevation in serum PSA level, and all participants underwent a transrectal ultrasound (TRUS) during which needle biopsies of prostate tissue were taken. Biopsied tissues were examined by a pathologist and the grade and stage of tumour tissue, if present, determined according to Gleason criteria and TMN classification, respectively [88,89]. Table 1 summarizes the characteristics and clinicopathological information for all subjects enrolled in the study.

### 4.2. Urine Collection and Processing

Voided urine samples (60–80 mL) were collected prior to TRUS-guided prostate biopsy and immediately following prostatic massage (post-digital rectal examination, post-DRE). Urine was collected into a sterile container containing urine preservative (Norgen Biotek Corp., Thorold, ON, Canada) and stored at room temperature (RT) to prevent the formation of precipitates. Within 48 h of collection, the samples were centrifuged in a swinging bucket centrifuge at 650× *g* for 10 min at RT to pellet the urine sediment/cell fraction. The resulting supernatants were then centrifuged at 10,000× *g* for 30 min. Sediment pellets and aliquoted 10,000× *g* urine supernatants were stored at −80 °C until further processing.

### 4.3. Isolation of Extracellular Vesicles Using the Vn96 Peptide

A 5 mL aliquot of 10,000× *g* urine supernatant from −80 °C storage was thawed at RT. The sample was then centrifuged at 17,000× *g* for 15 min at RT. An optimized amount of the Vn96 synthetic peptide (30 µg per 1 mL urine; New England Peptide Inc., Gardner, MA, USA) was added to the 17,000× *g* urine supernatant. The sample was incubated at RT with end-over-end rotation for 2 h followed by centrifugation at 17,000× *g* for 15 min at RT to pellet Vn96-EV complexes. After removal of supernatant and a wash with phosphate buffered saline (PBS), the Vn96-EV pellet was resuspended in 100 µL PBS and stored at −80 °C.

### 4.4. RNA Extraction from Urinary EVs

RNA was isolated from Vn96-isolated EVs using the RNeasy Plus Micro Kit (QIAGEN, Valencia, CA, USA) according to the manufacturer’s modified protocol for isolation of total RNA, including small RNA. In brief, 350 µL of Buffer RLT (with 10 µL β-mercaptoethanol per mL) was added to each suspension to lyse EVs. The lysate was then passed through a gDNA Eliminator spin column to remove DNA. Ethanol was added to the column flow-through and the sample applied to an RNeasy MinElute spin column with RNA-binding silica membrane. Following washes to remove contaminants, the RNA was eluted with 30 µL of nuclease-free water. The eluted RNA was stored at −80 °C.

### 4.5. Reverse Transcription Quantitative Polymerase Chain Reaction (RT-qPCR)

Vn96-isolated EV RNAs were quantified and profiled using a TapeStation system with High Sensitivity R6K ScreenTape (Agilent, Santa Clara, CA, USA). Synthesis of cDNA was performed using Superscript™ III Reverse Transcriptase (Invitrogen Corp., Grand Island, NY, USA) following the manufacturer’s instructions. For each sample, 10 µL of RNA was mixed with oligo(dT)_20_ and random primers (hexamers), dNTP mix and nuclease-free water to 13 µL. Following heating of the mixture at 65 °C for 5 min and incubation on ice for at least 3 min, a master mix comprising of 5× First-Strand buffer, 0.1 M DTT, RNaseOUT and SuperScript III Reverse Transcriptase was added on ice to achieve a final volume of 20 µL. The reverse transcription was performed using a thermal cycler protocol consisting of 10 min at 25 °C followed by 90 min at 50 °C. The enzyme was inactivated by heating at 70 °C for 15 min.

Multiplex pre-amplification of the target mRNAs was performed via PCR using iTaq™ DNA Polymerase (Bio-Rad Laboratories Inc., Hercules, CA, USA). A total of 6.25 µL of each cDNA sample was combined with DNA polymerase mix (containing buffer, polymerase, MgCl_2_ and dNTPs) and 5.5 µL of pooled forward and reverse primers in a total PCR reaction volume of 25 µL. The pool of mRNA-specific primers included those for *PCA3*, *KLK3*, *FOLH1*, *LTPB4*, *NELL2*, *HPN*, *XBP1*, *ITSN1*, *GSTM4*, *CFD*, *GOLM1*, *ANXA3*, *CD24*, *TMPRSS2-ERG*, *PSCA*, *SLC45A3*, and *ACTB* (primer sequences are provided in Supplementary Table S1). Primers were synthesized by IDT (Integrated DNA Technologies, Coralville, IA, USA). Primers were used in the pre-amplification reaction at a final concentration of 30 nM to 60 nM each. Fifteen pre-amplification cycles were employed using the following thermal cycling program: 1 cycle of 3 min at 95 °C; 15 cycles of 30 s at 95 °C, followed by 30 s at 60 °C (annealing), and 30 s at 72 °C (extension).

Levels of each mRNA were measured by quantitative PCR (qPCR) using iQ™ SYBR^®^ Green Supermix (Bio-Rad Laboratories Inc., Hercules, CA, USA) and a Mastercycler^®^ RealPlex^2^ instrument (Eppendorf Canada Ltd., Mississauga, ON, Canada). Each pre-amplified cDNA was diluted 1:5 with nuclease-free water and 5 µL of the dilution was combined with 10 µL Supermix, 4 µL of forward and reverse primers at 100 nM to 600 nM final concentration each, depending on the primer pair, and nuclease-free water to obtain a 20 µL final reaction volume. Primer sequences are provided in Supplementary Table S3. Each sample was analyzed in triplicate. Samples with quantification cycle (*Cq*) > 35 were deemed to be nonspecific amplification. The following qPCR cycling conditions were used: 1 cycle of 3 min at 95 °C; 40 cycles of 30 s at 95 °C, followed by 30 s at 60 °C (annealing), and 30 s at 72 °C (extension); and 1 cycle of 5 min at 72 °C. Melt curve analysis was performed to confirm product specificity.

Quantitative PCR efficiencies (*E*) for each mRNA were calculated using the following formula:

*E* = 10^(−1/slope)^. (1)

To correct for run-to-run differences, the efficiency-corrected relative expression (RE) of each target mRNA to an exogenous control (a pool of LNCaP and DU145 cell line cDNA) was then calculated. The calculation was based on *E* and the *Cq* deviation of each test sample versus the control sample, and the following equation was used [90]:

RE = (*E*_target_)^Δ*Cq*^_target(control-sample)_ (2)

*PCA3* values were calculated using the ratio of *PCA3* expression to *KLK3* expression in the formula:

*PCA3* = RE_*PCA3*_/RE_*KLK3*_. (3)

Values for reference-free mRNA biomarker panels were derived using the average (geometric mean) of the relative expression results (to the exogenous control) for overexpressed mRNAs divided by the average of the relative expression results for underexpressed mRNAs within the panel (eliminating the need for normalization to endogenous reference mRNAs). Messenger RNAs were previously shown to be either overexpressed or underexpressed in prostate tumour tissue relative to normal tissue [37]. Hence, reference-free mRNA panel values were calculated using the following equation:

Value = overexpressed geomean (RE_χ1_,…,RE_χη_)/underexpressed geomean(RE_χ1_,…,RE_χη_), where χ = individual mRNA of interest. (4)

### 4.6. miRNA Detection and Quantification

For detection of miRNAs, total RNA was reverse transcribed using the miRNA 1st-Strand cDNA Synthesis Kit (Agilent Technologies, Inc., Santa Clara, CA, USA) and following the manufacturer’s instructions. miRNAs present in each sample were first polyadenylated in a poly(A) polymerase reaction. An amount of 10 µL total RNA was mixed with 4 µL of 5× Poly(A) Tailing Buffer, 1 µL rATP, 1 µL of Poly(A) Polymerase, and nuclease-free water to a 20 µL final reaction volume. The mixture was incubated at 37 °C for 30 min and adenylation then terminated by incubation at 95 °C for 5 min. Ten microlitres of the polyadenylated RNA was then combined with 2 µL 10× AffinityScript RT buffer, 0.8 µL dNTP mix, 1 µL RT adaptor primer, 1 µL AffinityScript RT/RNase Block, and nuclease-free water to 20 µL. The mixture was incubated for 5 min at 55 °C, followed by 15 min at 25 °C, and then 30 min at 42 °C. Reverse transcription was terminated by incubation 5 min at 95 °C.

Quantification of miRNA targets was performed by qPCR using iQ™ SYBR^®^ Green Supermix (Bio-Rad Laboratories Inc., Hercules, CA, USA) and a Mastercycler^®^ RealPlex^2^ instrument (Eppendorf Canada Ltd., Mississauga, ON, Canada). A volume of 5 µL of cDNA was combined with 10 µL 2× SYBR Green Supermix, 2 µL of miRNA-specific forward primer to 300 nM final concentration, 2 µL Universal Reverse Primer (Agilent Technologies, Inc., Santa Clara, CA, USA) to 100 nM final concentration, and nuclease-free water to a 20 µL total reaction volume. The forward primer sequences for miR-574-3p, miR-375-3p, miR-141-3p, miR-21-3p, and *SNORD44* (used as a reference small mRNA) are provided in Supplementary Table S4. Each sample was analyzed in triplicate. Samples with quantification cycle (*Cq*) > 35 were deemed to be nonspecific amplification. The following qPCR cycling conditions were used: 1 cycle of 3 min at 95 °C; 40 cycles of 15 s at 95 °C, followed by 30 s at 60 °C, and 20 s at 72 °C. Melt curve analysis was performed to confirm product specificity.

Quantitative PCR efficiencies (*E*) for each miRNA were calculated as for mRNAs using formula 1. To correct for run-to-run differences, the relative expression (RE) of each target miRNA to that of an exogenous control sample was determined. The control sample was a pool of LNCaP and DU145 total RNA that was reverse transcribed alongside test samples using the same miRNA 1st-Strand cDNA Synthesis Kit. The calculation was based on the *Cq* difference of each test and control sample and used formula 2. The expression of each miRNA was then normalized using *SNORD44* as the endogenous reference. Relative quantification (RQ) of target miRNA (ϒ) to *SNORD44* was calculated using the formula:

RQ = RE_ϒ_/RE*_SNORD44_* × 10. (5)

Values for miRNA panels were calculated as the geometric mean of relative quantification results for the miRNAs in the panel using the formula:

Value = geomean(RQ_ϒ1_,…,RQ_ϒ__η_). (6)

### 4.7. Statistical Analysis

Univariate logistic regression was used to assess the predictive ability of each mRNA, miRNA, and mRNA or miRNA panel to distinguish prostate cancer from benign control groups. To derive optimized reference-free mRNA panels, the impact of removal of one mRNA at a time on the predictive ability of the panel (odds ratio and significance) was assessed. Multivariable logistic regression was used to estimate the additive value of combining the optimized reference-free mRNA panel with the miRNA panel, *PCA3* mRNA level and clinical characteristics. The diagnostic performance of each biomarker panel and model was evaluated using receiver operator characteristic (ROC) curve analysis with the calculated area under the curve (AUC) used to define diagnostic accuracy. Where noted, sensitivity and specificity were obtained using Youden Index analysis to determine the optimal discrimination threshold. Mann-Whitney U test was used to compare scores of the various panels and models between prostate cancer and benign control subject groups. *p* values < 0.05 were considered to indicate statistically significant differences. All differences denoted by asterisks in figures were statistically significant (* *p* < 0.05, ** *p* < 0.01, and *** *p* < 0.001). Statistical analyses were performed using GraphPad Prism 8 (GraphPad Software Inc., San Diego, CA, USA). ROC curve analyses were performed using the easyROC interactive web-tool (version 1.3.1, http://www.biosoft.hacettepe.edu.tr/easyROC/, accessed on 04/11/2020) [91]. Box plots were created using the BoxPlotR web tool (http://shiny.chemgrid.org/boxplotr/, accessed on 04/11/2020) [92].

## 5. Conclusions

In summary, we have demonstrated that our Vn96 technology is a simple and efficient peptide-based affinity method to isolate urinary EVs containing diagnostically relevant cargo for prostate cancer. Although requiring further testing and validation, the combination of our seven mRNA and two miRNA panels shows promising diagnostic value, particularly when used in conjunction with current clinical parameters. Liquid biopsy methods have garnered much attention as non- or minimally-invasive tools to discover and measure biomarkers, which may help guide treatment decisions and therapies tailored to individualized patient care. In the field of prostate cancer management, a number of needs in the areas of diagnosis (guiding biopsy and improving the specificity of sPSA), prognosis (discerning indolent from aggressive tumours), and prediction (guidance for treatment selection) remain unmet. A liquid biopsy platform utilizing Vn96 peptide-mediated isolation of EVs from urine may allow for the discovery and clinical implementation of sensitive and specific biomarkers with applications in these areas of prostate cancer management.

## Figures and Tables

**Table 1:** Clinicopathological characteristics of patients enrolled in the study.

	Patient Group		
	Total	Benign Controls (BC)	Patients with Prostate Cancer (PrCa)
Number (*n*)	56	28	28
Age (years)			
Mean (±SD)	67.6 (±6.8)	67.3 (±7.4)	68.1 (±6.3)
Range	48–79	50–79	48–79
Serum PSA (ng/mL)			
Mean (±SD)	4.6 (±2.6)	4.5 (±3.0)	4.8 (±2.1)
Range	0.6–14.7	0.6–14.7	0.9–9.1
0–4 ng/mL	22 (39%)	13 (46%)	9 (32%)
4–10 ng/mL	32 (57%)	13 (46%)	19 (68%)
>10 ng/mL	1(1.8%)	1 (3.6%)	0
Unknown	1	1	0
Other prostate conditions			
BPH *	16	7	9
PIN †	13	5	8
Both BPH and PIN	7	1	6
Nodule(s)	11	5	6
LUTS ††	6	1	5
Firm to touch	13	7	6
Increased volume	22	13	9
Lobe Asymmetry	11	3	8
Gleason grade at diagnosis			
≤6 (3 + 3)	NA	NA	21
7 (3 + 4)	NA	NA	4
7 (4 + 3) or higher	NA	NA	3
Clinical Stage			
T1	NA	NA	9
T2	NA	NA	11
T3	NA	NA	1
Not Available	NA	NA	7

* BPH, benign prostatic hyperplasia; † PIN, prostatic intraepithelial neoplasia; †† LUTS, lower urinary tract symptoms; NA, Not Applicable. Age and sPSA level were not significantly different between BC and PrCa groups with *p* values of 0.5823 and 0.2756, respectively.

**Table 2:** Predictive and diagnostic performance of reference-free mRNA biomarker panels for prostate cancer using Vn96-isolated EVs.

mRNA Panel Variable	Logistic Regression Analysis		ROC Curve Analysis	
	OR (95% CI)	*p* Value	AUC (95% CI)	*p* Value
**8 mRNA Panel**	1.0396 (1.0062, 1.0741)	0.0199	0.695 (0.553, 0.837)	0.0071
*LTBP4* and *NELL2* removed	1.2386 (1.0360, 1.4809)	0.0189	0.694 (0.555, 0.832)	0.0061
**5 mRNA Panels**				
*FOLH1* removed	1.0488 (0.9439, 1.1655)	0.3755	0.651 (0.502, 0.799)	0.0473
*HPN* removed	1.4259 (1.0146, 2.0040)	0.041	0.667 (0.523, 0.811)	0.023
*XBP1* removed	1.1272 (1.0416, 1.2199)	0.003	0.761 (0.636, 0.887)	<0.0001
*ITSN1* removed	1.0154 (0.9616, 1.0722)	0.5817	0.67 (0.525, 0.814)	0.0212
*GSTM4* removed	1.2439 (1.0482, 1.4762)	0.0124	0.751 (0.623, 0.880)	0.0001
*CFD* removed	1.1995 (0.9935, 1.4481)	0.0584	0.662 (0.519, 0.805)	0.026
**4 mRNA Panel**				
(*FOLH1*, *HPN*, *ITSN1*, *CFD*)	1.1359 (1.0479, 1.2313)	0.002	0.798 (0.681, 0.916)	<0.0001

**Table 3:** Performance of urinary Vn96-isolated EV reference-free mRNA biomarker panels for prostate cancer prediction and diagnosis.

mRNA Panel Variable	Logistic Regression Analysis		ROC Curve Analysis	
	OR (95% CI)	*p* Value	AUC (95% CI)	*p* Value
**4 mRNA Panel**				
(*FOLH1, HPN, ITSN1, CFD*)	1.1359 (1.0479, 1.2313)	0.002	0.798 (0.681, 0.916)	6.67 × 10^−7^
**New 6 mRNA Panel**				
(*GOLM1, ANXA3, CD24, TMPRSS2-ERG, PSCA, SLC45A3*)	4.4649 (1.3686, 14.5668)	0.0131	0.725 (0.585, 0.865)	1.62 × 10^−3^
**Combined 10 mRNA Panel**	1.6359 (1.1808, 2.2663)	0.0031	0.759 (0.630, 0.888)	8.49 × 10^−5^
*GOLM1* removed	1.5596 (1.1723, 2.0748)	0.0023	0.797 (0.677, 0.917)	1.19 × 10^−6^
*ANXA3* removed	1.0827 (0.9981, 1.1745)	0.0557	0.709 (0.570, 0.849)	3.33 × 10^−3^
*CD24* removed	1.7905 (1.2128, 2.6433)	0.0034	0.769 (0.643, 0.896)	3.08 × 10^−5^
*TMPRSS2-ERG* removed	1.5597 (1.0128, 2.4017)	0.0436	0.704 (0.563, 0.845)	4.52 × 10^−3^
*PSCA* removed	1.5355 (1.1665, 2.0213)	0.0022	0.781 (0.656, 0.905)	1.03 × 10^−5^
*SLC45A3* removed	1.1223 (0.9776, 1.2885)	0.1013	0.723 (0.584, 0.862)	1.66 × 10^−3^
*FOLH1* removed	1.0836 (0.9653, 1.2164)	0.1733	0.699 (0.558, 0.840)	5.54 × 10^−3^
*HPN* removed	1.5006 (1.1264, 1.9991)	0.0055	0.741 (0.608, 0.875)	4.00 × 10^−4^
*ITSN1* removed	2.1580 (1.2820, 3.6325)	0.0038	0.749 (0.617, 0.880)	2.05 × 10^−4^
*CFD* removed	3.1347 (1.5581, 6.3064)	0.0014	0.787 (0.663, 0.911)	5.82 × 10^−6^
**7 mRNA Panel**				
(*ANXA3, CD24, TMPRSS2-ERG, SLC45A3, FOLH1, HPN, ITSN1*)	2.2371 (1.4036, 3.5656)	0.0007	0.825 (0.710, 0.941)	3.18 × 10^−8^

**Table 4:** Performance of urinary Vn96-isolated EV miRNA biomarkers and combined mRNA + miRNA model for prostate cancer prediction and diagnosis.

miRNA or Combined Variable	Logistic Regression Analysis		ROC Curve Analysis	
Univariate	OR (95% CI)	*p* Value	AUC (95% CI)	*p* Value
miR-141-3p	1.6604 (1.0373, 2.6578)	0.0346	0.645 (0.492, 0.797)	0.0629
miR-375-3p	1.1049 (1.0130, 1.2051)	0.0243	0.744 (0.603, 0.885)	0.0007
miR-574-3p	1.7572 (1.0882, 2.8373)	0.0211	0.733 (0.599, 0.866)	0.0006
miR-21-3p	1.2562 (1.0154, 1.5540)	0.0357	0.698 (0.553, 0.843)	0.0073
4 miRNA Panel	1.5012 (1.0761, 2.0942)	0.0168	0.719 (0.574, 0.865)	0.0031
3 miRNA Panel (miR-141-3p, miR-375-3p, miR-574-3p)	1.4851 (1.0815, 2.0393)	0.0145	0.704 (0.559, 0.849)	0.0059
3 miRNA Panel (miR-375-3p, miR-574-3p, miR-21-3p)	1.3990 (1.0750, 1.8206)	0.0125	0.737 (0.597, 0.876)	0.0009
2 miRNA Panel (miR-375-3p, miR-574-3p)	1.3390 (1.0592, 1.6928)	0.0147	0.744 (0.607, 0.880)	0.0005
**Multivariable**				
7 Gene Score + 2 miRNA Model	NA	NA	0.843 (0.722, 0.964)	2.55 × 10^−8^
7 mRNA Panel	1.8463 (1.1375, 2.9968)	0.0131	NA	NA
2 miRNA Panel	1.1822 (0.9581, 1.4587)	0.1822	NA	NA

NA, Not Applicable.

**Table 5:** Performance of multivariable models combining urinary Vn96-isolated EV mRNAs and miRNAs with clinical characteristics for prostate cancer prediction and diagnosis.

Variable	Logistic Regression Analysis		ROC Curve Analysis	
Univariate	OR (95% CI)	*p* Value	AUC (95% CI)	*p* Value
Urinary Vn96-EV *PCA3*	1.0960 (1.0298, 1.1665)	0.0039	0.816 (0.708, 0.925)	1.20 × 10^−8^
Age at collection	1.0181 (0.9420, 1.1004)	0.6509	0.543 (0.387, 0.700)	0.5864
Serum PSA at collection	1.0742 (0.8671, 1.3308)	0.5124	0.594 (0.440, 0.749)	0.2303
Prostate Volume at collection	0.9726 (0.9421, 1.0041)	0.0874	0.656 (0.508, 0.805)	0.0391
DRE Result	0.8627 (0.2972, 2.5041)	0.7860	0.518 (0.387, 0.649)	0.7895
**Multivariable**				
**Model 1 (SOC ^†^ Only)**	NA	NA	0.718 (0.583, 0.853)	0.0016
Age	1.0746 (0.9785, 1.1802)	0.1322	NA	NA
Serum PSA	1.2333 (0.9385, 1.6209)	0.1325	NA	NA
Prostate Volume	0.9427 (0.9001, 0.9873)	0.0123	NA	NA
DRE Result	0.4799 (0.1327, 1.7351)	0.2629	NA	NA
**Model 2 ^††^**	NA	NA	0.901 (0.811, 0.990)	1.75 × 10^−18^
7 mRNA Panel	1.7053 (1.0026, 2.9005)	0.0489	NA	NA
2 miRNA Panel	1.1670 (0.9429, 1.4445)	0.1088	NA	NA
*PCA3* Value	1.0615 (1.0047, 1.1216)	0.0335	NA	NA
**Model 3 ^†††^**	NA	NA	0.955 (0.909, 1.002)	2.27 × 10^−82^
7 mRNA Panel	1.4312 (0.7581, 2.7021)	0.2688	NA	NA
2 miRNA Panel	1.4024 (0.9767, 2.0138)	0.0669	NA	NA
*PCA3* Value	1.0810 (0.9984, 1.1705)	0.0548	NA	NA
Age	1.0921 (0.8962, 1.3309)	0.3823	NA	NA
Serum PSA	1.5220 (0.8605, 2.6921)	0.1489	NA	NA
Prostate Volume	0.8741 (0.7764, 0.9841)	0.0261	NA	NA
DRE Results	0.1393 (0.0117, 1.6573)	0.1187	NA	NA

NA, Not Applicable; ^†^ SOC, Standard of Care clinical characteristics; NA, ^††^ Model 2 combines Vn96-isolated EV 7 mRNA panel, 2 miRNA panel and *PCA3* value; ^†††^ Model 3 combines Vn96-isolated EV seven mRNA panel, two miRNA panel and *PCA3* value with SOC clinical characteristics.

## Supplementary Materials

The following are available online at https://www.mdpi.com/1422-0067/21/21/8330/s1.

## Abbreviations

*ACTB*	Actin Beta
*ANXA3*	Annexin A3
AUC	Area Under the Curve
BC	Benign Control
BPH	Benign Prostate Hyperplasia
*CFD*	Complement Factor D
DNA	Deoxyribonucleic acid
dNTP	Deoxynucleoside triphosphate
DRE	Digital Rectal Examination
DTT	Dithiothreitol
*ERG*	Erythroblast Transformation-Specific (ETS) Transcription Factor ERG
EV	Extracellular Vesicle
*FOLH1*	Folate Hydrolase 1
*GOLM1*	Golgi Membrane Protein 1
*GOLPH2*	Golgi phosphoprotein 2
*GSTM4*	Glutathione S-Transferase Mu 4
*HPN*	Hepsin
Hsp/c70	Heat shock protein/cognate 70
*ITSN1*	Intersectin 1
PIN	Prostatic Intraepithelial Neoplasia
KLK3	Kallikrein Related Peptidase 3
*LTBP4*	Latent Transforming Growth Factor Beta Binding Protein 4
mRNA	Messenger ribonucleic acid
*NELL2*	Neural EGFL Like 2
PBS	Phosphate-buffered saline
*PCA3*	Prostate Cancer Associated 3
PDCD6IP	Programmed Cell Death 6 Interacting Protein
PrCa	Prostate Cancer
*PSCA*	Prostate Stem Cell Antigen
qPCR	Quantitative Polymerase Chain Reaction
ROC	Receiver Operator Characteristic
SDS-PAGE	Sodium dodecyl sulphate-polyacrylamide gel electrophoresis
*SLC45A3*	Solute Carrier Family 45 Member 3
*SNORD44*	Small Nucleolar RNA, C/D Box 44
*SPINK1*	Serine Peptidase Inhibitor Kazal Type 1
sPSA	Serum Prostate Specific Antigen
TMN	Tumour, nodes, metastasis
*TMPRSS2*	Transmembrane Serine Protease 2
TRUS	Transrectal Ultrasound
*XBP1*	X-Box Binding Protein 1
