# Supplementary material for: Affinity Captured Urinary Extracellular Vesicles Provide mRNA and miRNA Biomarkers for Improved Accuracy of Prostate Cancer Detection: A Pilot Study
Source: Int J Mol Sci. 2020 Nov 6;21(21):8330. doi: 10.3390/ijms21218330 (PMC7664192; doi:10.3390/ijms21218330)
Supplement: Supplementary file 1 [file ijms-21-08330-s001.zip › Supplemental Figure S1.docx]

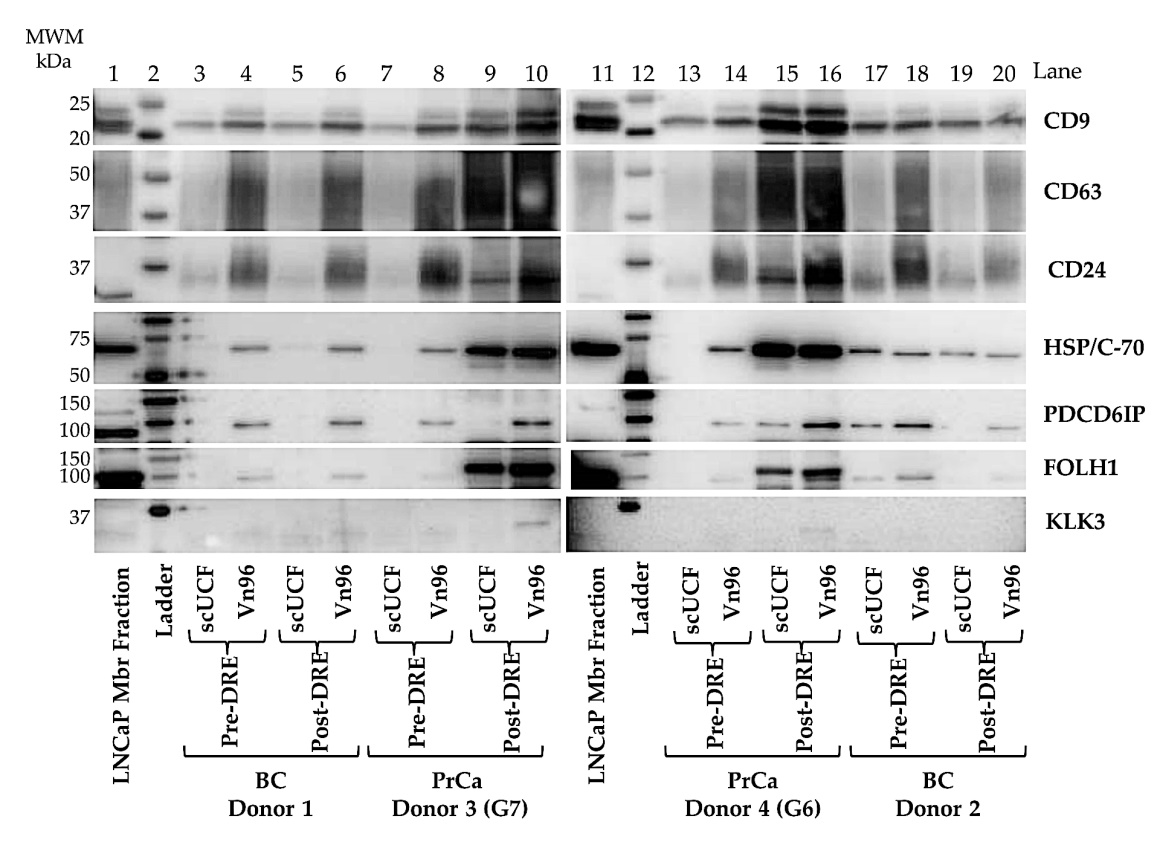


**Supplemental Figure S1.** *The Vn96 peptide isolates EVs from urine.* Western blot analysis of proteins extracted from EVs isolated with either the Vn96 peptide or scUCF, in parallel, from equal volumes of pre- and post-DRE urine. The entire amount of material extracted from 5 mL of urine by either Vn96 peptide or scUCF EV isolation method was suspended in the same volume of Laemmli buffer for each sample and equal volumes of solubilized sample loaded into each lane. Donor 1 and Donor 2 samples were from patients whose biopsy results were negative (Benign Control; BC), while Donor 3 and Donor 4 samples were from patients with positive biopsy results (Prostate Cancer; PrCa). The Gleason scores for Donor 3 and Donor 4 were 7 and 6, respectively. Urinary EVs contained canonical EV protein markers (CD9, CD63, CD24, Hsp/c-70, PDCD6IP) and the prostate-specific markers FOLH1 (PSMA) and KLK3 (PSA).

Isolation of EVs from urine using the Vn96 peptide

We wished to compare the efficiency of EV isolation from urine using Vn96 peptide versus the most commonly used method, ultracentrifugation. Urine from four study participants was used: two patients whose biopsy results were positive for prostate cancer (PrCa) and two patients whose biopsy results were negative (benign control, BC). EVs were isolated in parallel from equal volumes of urine collected both prior to DRE (pre-DRE) and immediately following DRE (post-DRE) using the Vn96 and sucrose cushion ultracentrifugation (scUCF) methods. EVs were assessed for canonical EV protein markers and prostate-specific protein markers by Western blot analyses (Figure S1). Urinary EVs captured with Vn96 contained common EV protein markers (CD9, CD63, CD24, Hsp/c70, PDCD6IP). These EV markers were all readily detected in Vn96-isolated EV preparations. In some cases they were not detected in the corresponding scUCF-isolated EV samples, particularly in the case of benign control and pre-DRE samples (e.g. HSP/C-70 lanes 7 and 13; PDCD6IP lanes 5, 7, 13 and 19). The prostate-specific protein marker FOLH1 was readily detected in both prostate cancer samples following DRE (post-DRE; lanes 7 and 8 compared to lanes 9 and 10, and lanes 13 and 14 compared to 15 and 16). Moreover, KLK3 was only detectable in post-DRE cancer samples where Vn96 was employed for EV isolation (lanes 10 and 16). Since DRE appeared to allow for enrichment of prostate-specific markers in urine samples of prostate cancer patients, we decided to continue with post-DRE urine for the remainder of the study.
