# Supplementary material for: Affinity Captured Urinary Extracellular Vesicles Provide mRNA and miRNA Biomarkers for Improved Accuracy of Prostate Cancer Detection: A Pilot Study
Source: Int J Mol Sci. 2020 Nov 6;21(21):8330. doi: 10.3390/ijms21218330 (PMC7664192; doi:10.3390/ijms21218330)
Supplement: Supplementary file 1 [file ijms-21-08330-s001.zip › Supplemental Figure S2.docx]

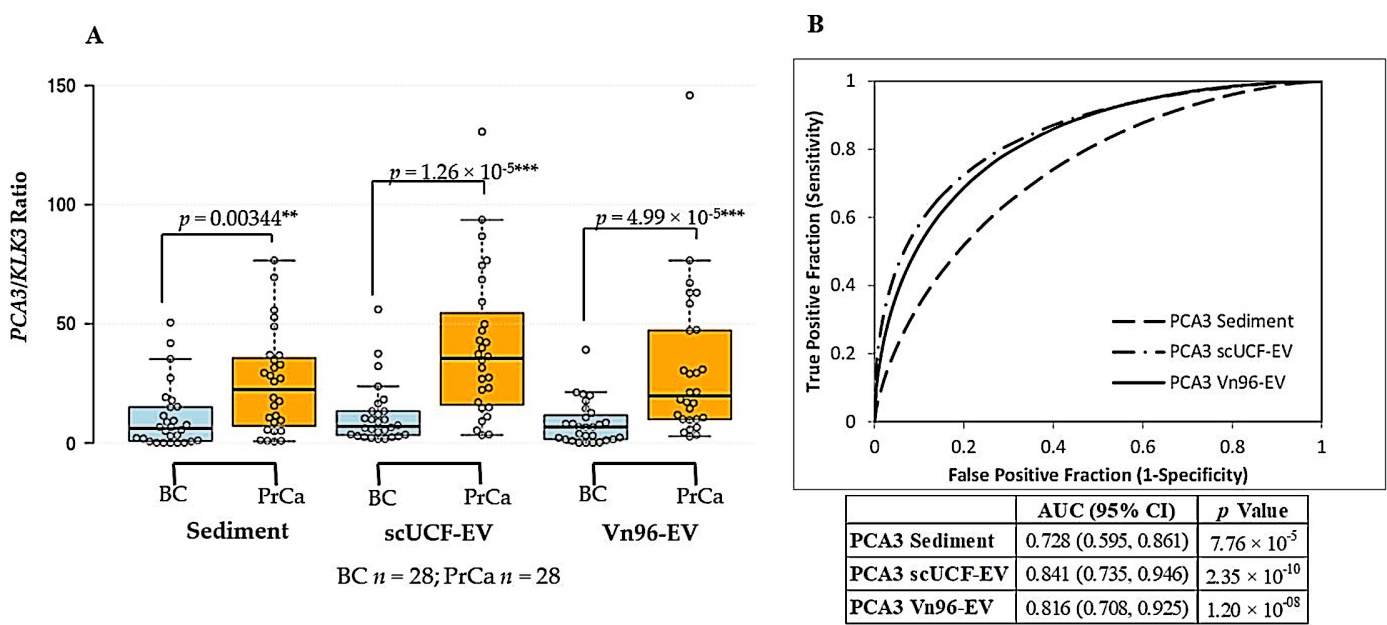


**Supplemental Figure S2.** *Comparison of PCA3/KLK3 ratios obtained using urine sediment RNA, scUCF-isolated EV RNA, and Vn96-isolated EV RNA.* **(A)** Box plot graphically depicting results of *PCA3/KLK3* ratios obtained using urine sediment RNA, scUCF-isolated EV RNA, and Vn96-isolated EV RNA. The central boxes represent the values from the lower to upper quartiles (25th to 75th percentiles). Lines within the boxes are the median values (50th percentile). Whiskers extending from the boxes indicate the minimum to maximum values obtained (excluding outliers, which are displayed as separate points). The Mann-Whitney U test was used to compare differences in values between Prostate Cancer (PrCa) and Benign Control (BC) groups. Resulting *p* values are provided above each paired group. Differences denoted by asterisks are statistically significant (* p < 0.05, ** p < 0.01, and *** p < 0.001). **(B)** ROC curve analysis showing discrimination of Prostate Cancer (PrCa) from Benign Control (BC) using *PCA3/KLK3* ratios obtained with urine sediment RNA, scUCF-isolated EV RNA, and Vn96-isolated EV RNA. AUC, 95% CI and *p* value are provided below the plot.

As a benchmark for our methods and to compare Vn96-isolated EVs to scUCF-isolated EVs and urine sediments for enrichment of prostate-specific mRNAs, we measured both *PCA3* and *KLK3* using RNA isolated from EV and sediment samples. *PCA3* values were calculated as a ratio of their expression to that of *KLK3*. We found a better distinction between benign control (*n* = 28) and prostate cancer (*n* = 28) groups using RNA derived from EVs (scUCF-isolated EVs: *P* value=1.26x10^-5^; Vn96-isolated EVs: *p* value=4.99x10^-5^) than RNA derived from sediment (*P* value=0.0034) (Figure S2A). Receiver operating characteristic (ROC) curve analyses of the data demonstrated that diagnostic accuracy of our *PCA3* test was improved using urinary EVs compared to urine sediment (Figure S2B). The area under the ROC curve (AUC) for sediment was 0.728 while the AUCs for scUCF-isolated EVs and Vn96-isolated EVs were comparable at 0.841 and 0.816, respectively. The use of EV-derived RNA improved both test sensitivity and specificity. At optimal thresholds, sensitivity using sediment was 71% (95% confidence interval, CI, 51-87%) compared to 82% (95% CI=63-94%) for either scUCF- or Vn96-isolated EVs. Specificity increased from 68% (95% CI=48-84%) for sediment to 79% (95% CI=59-92%) for scUCF-isolated EVs and 71% (95% CI=51-87%) for Vn96-isolated EVs. These results show that urinary EVs contain prostate-specific and diagnostically relevant RNAs and that our Vn96 peptide method for EV isolation performs comparably to ultracentrifugation for isolation of the clinically-relevant EV population.
