# Supplementary material for: Affinity Captured Urinary Extracellular Vesicles Provide mRNA and miRNA Biomarkers for Improved Accuracy of Prostate Cancer Detection: A Pilot Study
Source: Int J Mol Sci. 2020 Nov 6;21(21):8330. doi: 10.3390/ijms21218330 (PMC7664192; doi:10.3390/ijms21218330)
Supplement: Supplementary file 1 [file ijms-21-08330-s001.zip › Supplementary Materials and Methods.docx]

**Supplementary Materials and Methods**

Isolation of Extracellular Vesicles using Sucrose Cushion Ultracentrifugation

Sucrose cushion ultracentrifugation (scUCF) was used to isolate EVs as previously described [32]. Briefly, 5 mL of 17 000 ×g urine supernatant was transferred to an ultracentrifugation tube. A Pasteur pipette was carefully inserted beneath the sample to layer 500 µL of 30% (w/v) sucrose solution in PBS at the bottom of the tube. The sample was centrifuged at 100,000 ×g for two hours using a Beckman Coulter ultracentrifuge equipped with a SW-40Ti rotor. The EV-containing sucrose cushion was aspirated carefully using a Pasteur pipette into a new ultracentrifuge tube, diluted to 10 mL with PBS and re-centrifuged at 100,000 ×g for 90 minutes. The supernatant was discarded, and the EV pellet resuspended in 100 µL of PBS and stored at -80°C.

Western Blotting

Urinary Vn96-isolated EV or scUCF-isolated EV complexes were solubilized in 4× Laemmli sample buffer (with or without reducing agent) and proteins separated by SDS-PAGE on 12% polyacrylamide gels. Western blotting was performed as described in a previous study [32]. Primary antibodies used included mouse anti-CD9 (clone ALB-6), anti-CD63 (clone MX-49.129.5), anti-Hsp/c70 (clone SPM254), anti-ALIX (PDCD6IP, clone 1A12), anti-FOLH1 (PSMA, clone Y-PSMA2), anti-KLK3 (PSA, clone A67-B/E3) and rabbit anti-CD24 (FL-80); all antibodies were purchased from Santa Cruz Biotechnology (Dallas, TX, USA). Secondary antibody was human-adsorbed donkey anti-mouse or anti-rabbit IgG-HRP (Santa Cruz Biotechnology). The membrane-enriched fraction of a whole cell lysate of the prostate cancer cell line LNCaP was used as a positive control.

RNA Extraction from Urinary Sediment

Urinary sediment pellet containing exfoliated cells was processed, within 2 weeks of collection, for RNA isolation using the Urine Exfoliated Cell RNA Purification Kit (Norgen Biotek Corp.) according to the manufacturer’s instructions. In brief, 350 µL of Lysis Solution (with 10 µL β-mercaptoethanol per mL) was added to the pellet to lyse cells. Ethanol was then added to the lysate and the solution loaded onto a spin column containing RNA-binding resin. An on-column DNA removal step was included in the protocol using Norgen’s RNase-Free DNase I Kit. Following washes, total RNA, including mRNA and miRNA, was eluted in 30 µL of Elution Solution. The eluted RNA was stored at -80°C.

RNA Extraction from Urinary EVs

RNA was isolated from the Vn96-isolated EVs or scUCF-isolated EVs using the RNeasy Plus Micro Kit (QIAGEN, Valencia, CA, USA) according to the manufacturer’s modified protocol for isolation of total RNA, including small RNA. In brief, 350 µL of Buffer RLT (with 10 µL β-mercaptoethanol per mL) was added to each suspension to lyse EVs. The lysate was then passed through a gDNA Eliminator spin column to remove DNA. Ethanol was added to the column flow-through and the sample applied to an RNeasy MinElute spin column with RNA-binding silica membrane. Following washes to remove contaminants, the RNA was eluted with 30 µL of nuclease-free water. The eluted RNA was stored at -80°C.
