# Supplementary material for: Affinity Captured Urinary Extracellular Vesicles Provide mRNA and miRNA Biomarkers for Improved Accuracy of Prostate Cancer Detection: A Pilot Study
Source: Int J Mol Sci. 2020 Nov 6;21(21):8330. doi: 10.3390/ijms21218330 (PMC7664192; doi:10.3390/ijms21218330)
Supplement: Supplementary file 1 [file ijms-21-08330-s001.zip › Supplementary Table S1.docx]

**Supplementary Table S1.** Predictive and diagnostic performance of reference-free mRNA biomarker panels for prostate cancer using urinary sediments, scUCF-isolated EVs and Vn96-isolated EVs.

| **mRNA Panel Variable** | **Sample** | **Logistic Regression Analysis** | | **ROC Curve Analysis** | |
| --- | --- | --- | --- | --- | --- |
|  |  | **OR (95% CI)** | ***p* Value** | **AUC (95% CI)** | ***p* Value** |
| **8 mRNA Panel** | Sediment | 1.103 (0.9183, 1.3249) | 0.2944 | 0.614 (0.464, 0.763) | 0.1376 |
|  | scUCF-EV | 1.005 (0.9920, 1.0192) | 0.425 | 0.505 (0.349, 0.661) | 0.9488 |
|  | Vn96-EV | 1.0396 (1.0062, 1.0741) | 0.0199 | 0.695 (0.553, 0.837) | 0.0071 |
| *LTBP4* and *NELL2* removed | Sediment | 1.4175 (0.9209, 2.1821) | 0.1129 | 0.631 (0.484, 0.779) | 0.0804 |
|  | scUCF-EV | 1.0953 (0.9636, 1.2451) | 0.1637 | 0.634 (0.483, 0.785) | 0.0812 |
|  | Vn96-EV | 1.2386 (1.0360, 1.4809) | 0.0189 | 0.694 (0.555, 0.832) | 0.0061 |
| **5 mRNA Panels** |  |  |  |  |  |
| *FOLH1* removed | Sediment | 1.0443 (0.8488, 1.2848) | 0.6821 | 0.589 (0.436, 0.743) | 0.2536 |
|  | scUCF-EV | 1.0373 (0.9457, 1.1379) | 0.4373 | 0.622 (0.469, 0.776) | 0.1187 |
|  | Vn96-EV | 1.0488 (0.9439, 1.1655) | 0.3755 | 0.651 (0.502, 0.799) | 0.0473 |
| *HPN* removed | Sediment | 1.1965 (0.6120, 2.3393) | 0.6 | 0.614 (0.462, 0.765) | 0.1423 |
|  | scUCF-EV | 1.2347 (0.9419, 1.6185) | 0.1269 | 0.612 (0.461, 0.763) | 0.1451 |
|  | Vn96-EV | 1.4259 (1.0146, 2.0040) | 0.041 | 0.667 (0.523, 0.811) | 0.023 |
| *XBP1* removed | Sediment | 1.3790 (0.9980, 1.9055) | 0.0514 | 0.654 (0.510, 0.799) | 0.0363 |
|  | scUCF-EV | 1.0507 (0.9969, 1.1074) | 0.0649 | 0.663 (0.517, 0.809) | 0.0285 |
|  | Vn96-EV | 1.1272 (1.0416, 1.2199) | 0.003 | 0.761 (0.636, 0.887) | <0.0001 |
| *ITSN1* removed | Sediment | 2.0139 (0.9628, 4.2126) | 0.063 | 0.631 (0.485, 0.778) | 0.0797 |
|  | scUCF-EV | 1.1534 (1.0001, 1.3301) | 0.0498 | 0.663 (0.517, 0.809) | 0.0283 |
|  | Vn96-EV | 1.0154 (0.9616, 1.0722) | 0.5817 | 0.67 (0.525, 0.814) | 0.0212 |
| *GSTM4* removed | Sediment | 0.9752 (0.8694, 1.0939) | 0.6679 | 0.661 (0.515, 0.806) | 0.0301 |
|  | scUCF-EV | 1.0154 (0.9769, 1.0555) | 0.4384 | 0.594 (0.440, 0.749) | 0.2299 |
|  | Vn96-EV | 1.2439 (1.0482, 1.4762) | 0.0124 | 0.751 (0.623, 0.880) | 0.0001 |
| *CFD* removed | Sediment | 1.0791 (0.8650, 1.3462) | 0.4999 | 0.580 (0.428, 0.733) | 0.3013 |
|  | scUCF-EV | 1.0296 (0.9555, 1.1093) | 0.4443 | 0.617 (0.466, 0.769) | 0.1289 |
|  | Vn96-EV | 1.1995 (0.9935, 1.4481) | 0.0584 | 0.662 (0.519, 0.805) | 0.026 |
|  |  |  |  |  |  |
| **4 mRNA Panel**  *(FOLH1, HPN, ITSN1, CFD)* | Sediment | 1.1975 (0.9767, 1.4681) | 0.083 | 0.676 (0.533, 0.819) | 0.0158 |
|  | scUCF-EV | 1.0108 (0.9885, 1.0336) | 0.3461 | 0.64 (0.494, 0.787) | 0.0603 |
|  | Vn96-EV | 1.1359 (1.0479, 1.2313) | 0.002 | 0.798 (0.681, 0.916) | <0.0001 |
