# Supplementary material for: Affinity Captured Urinary Extracellular Vesicles Provide mRNA and miRNA Biomarkers for Improved Accuracy of Prostate Cancer Detection: A Pilot Study
Source: Int J Mol Sci. 2020 Nov 6;21(21):8330. doi: 10.3390/ijms21218330 (PMC7664192; doi:10.3390/ijms21218330)
Supplement: Supplementary file 1 [file ijms-21-08330-s001.zip › Supplementary Table S2.docx]

**Supplementary Table S2.** miRNA biomarkers evaluated by RT-qPCR using RNA extracted from Vn96-isolated EVs.

| **miRNA** | **Detected, discriminatory** | **Detected, not discriminatory** | **Not Detected** |
| --- | --- | --- | --- |
| miR-107 |  | ● |  |
| miR-200b |  | ● |  |
| miR-21 | ● |  |  |
| miR-205 |  |  | ● |
| miR-625 |  |  | ● |
| miR-483 |  |  | ● |
| miR-574 | ● |  |  |
| miR-301a |  |  | ● |
| miR-34a |  | ● |  |
| miR-19b |  | ● |  |
| miR-16 |  | ● |  |
| miR-375 | ● |  |  |
| miR-141 | ● |  |  |
