# Supplementary material for: Affinity Captured Urinary Extracellular Vesicles Provide mRNA and miRNA Biomarkers for Improved Accuracy of Prostate Cancer Detection: A Pilot Study
Source: Int J Mol Sci. 2020 Nov 6;21(21):8330. doi: 10.3390/ijms21218330 (PMC7664192; doi:10.3390/ijms21218330)
Supplement: Supplementary file 1 [file ijms-21-08330-s001.zip › Supplementary Table S3.docx]

**Supplementary Table S3**: List of primers used for mRNA RT-qPCR

| **Gene Target** | **Forward Primer Sequence** | **Reverse Primer Sequence** |
| --- | --- | --- |
| ***TMPRSS2-ERG*** | 5’-TAG GCG CGA GCT AAG CAG GAG-3’ | 5’-GTA GGC ACA CTC AAA CAA CGA CTG G-3’ |
| ***GOLM1*** | 5’-GCA GCT TGA CAA AAT CCA GTC-3’ | 5’-AGG GTC TTT AAC TGG TCT TGC-3’ |
| ***CD24*** | 5’-CCA AAT CCA ACT AAT GCC ACC-3’ | 5’-ACG TTT CTT GGC CTG AGT C-3’ |
| ***ANXA3*** | 5’-GTT TGT TCG CAG TTT ACT CGC-3’ | 5’-CTG ATG GGC TAA AGT CTG GAT-3’ |
| ***PSCA*** | 5’-CTG TTG ATG GCA GGC TTG GC-3’ | 5’-GGC CAA CTG CGC GGA TG-3’ |
| ***SLC45A3*** | 5’-GCC AGG ATC TGA GTG ATG AGA-3’ | 5’-GTT CAG GCA CTC CAG AAC TG-3’ |
| ***PCA3*** | 5’-TGG GAA GGA CCT GAT GAT ACA-3’ | 5’-CCC AGG GAT CTC TGT GCT T-3’ |
| ***KLK3*** | 5’-TGC CCA CTG CAT CAG GAA CA-3’ | 5’-CAT CAC CTG GCC TGA GGA ATC-3’ |
| ***FOLH1*** | 5'-TCC ATT AGG GTT ACC AGA CAGG-3' | 5'-GCA ATG ACT CCC CTG CAT AC-3' |
| ***HPN*** | 5'-GCG AGG AGA ACA GCA ACG-3' | 5'-ACA GGC TGG ATG TAT TCT GTG A-3' |
| ***ITSN1*** | 5'-TGG AAC TGC TGG GAA AACA-3' | 5'-GGC GGT GTA TGA GGC AAT A-3' |
| ***GSTM4*** | 5'-ACT TCA TCT CCC GCT TTG AG-3' | 5'-GAG GAA GCG GCT GGA CTT-3' |
| ***CFD*** | 5'-AAG CGC CTG TAC GAC GTG-3' | 5'-GAC AGC TGT AGC AGC AGG A-3' |
| ***ACTB*** | 5'-GCA CCC AGC ACA ATG AAG A-3' | 5'-CGA TCC ACA CGG AGT ACT TG-3' |
|  |  |  |
|  |  |  |
