# Supplementary material for: Affinity Captured Urinary Extracellular Vesicles Provide mRNA and miRNA Biomarkers for Improved Accuracy of Prostate Cancer Detection: A Pilot Study
Source: Int J Mol Sci. 2020 Nov 6;21(21):8330. doi: 10.3390/ijms21218330 (PMC7664192; doi:10.3390/ijms21218330)
Supplement: Supplementary file 1 [file ijms-21-08330-s001.zip › Supplementary Table S4.docx]

**Supplementary Table S4**: List of primers used for miRNA RT-qPCR

| **miRNA Target** | **Mature Sequence (miRBase)** | **Forward Primer Sequence (for qPCR)*** | |
| --- | --- | --- | --- |
|  |  |  | |
| **hsa-miR-141-3p** | 5’-UAA CAC UGU CUG GUA AAG AUG G-3’ | 5’-TAA CAC TGT CTG GTA AAG ATG G-3’ | |
|  |  |  | |
| **hsa-miR-375-3p** | 5’-UUU GUU CGU UCG GCU CGC GUG A-3’ | 5’-TTT GTT CGT TCG GCT CGC GTG A-3’ | |
|  |  |  | |
| **hsa-miR-574-3p** | 5’-CAC GCU CAU GCA CAC ACC CAC A-3’ | 5’-CAC GCT CAT GCA CAC ACC CAC A-3’ | |
|  |  |  | |
| **hsa-miR-21-3p** | 5’-CAA CAC CAG UCG AUG GGC UGU-3’ | 5’-CAA CAC CAG TCG ATG GGC TGT-3’ | |
|  |  |  | |
| ***SNORD44*** | 5'-CCTGGATGATGATAAGCAAATGCTGA  CTGAACATGAAGGTCTTAATTAGCTCTAACTGACT-3' | 5'-CTG GAT GAT GAT AAG CAA ATG C-3' | |
| *miRNA-specific forward primers were paired with a Universal Reverse Primer for amplification | | | |
| of the target sequence | | |  |
