# Supplementary figures and images for: Affinity Captured Urinary Extracellular Vesicles Provide mRNA and miRNA Biomarkers for Improved Accuracy of Prostate Cancer Detection: A Pilot Study
Source: Int J Mol Sci. 2020 Nov 6;21(21):8330. doi: 10.3390/ijms21218330 (PMC7664192; doi:10.3390/ijms21218330)

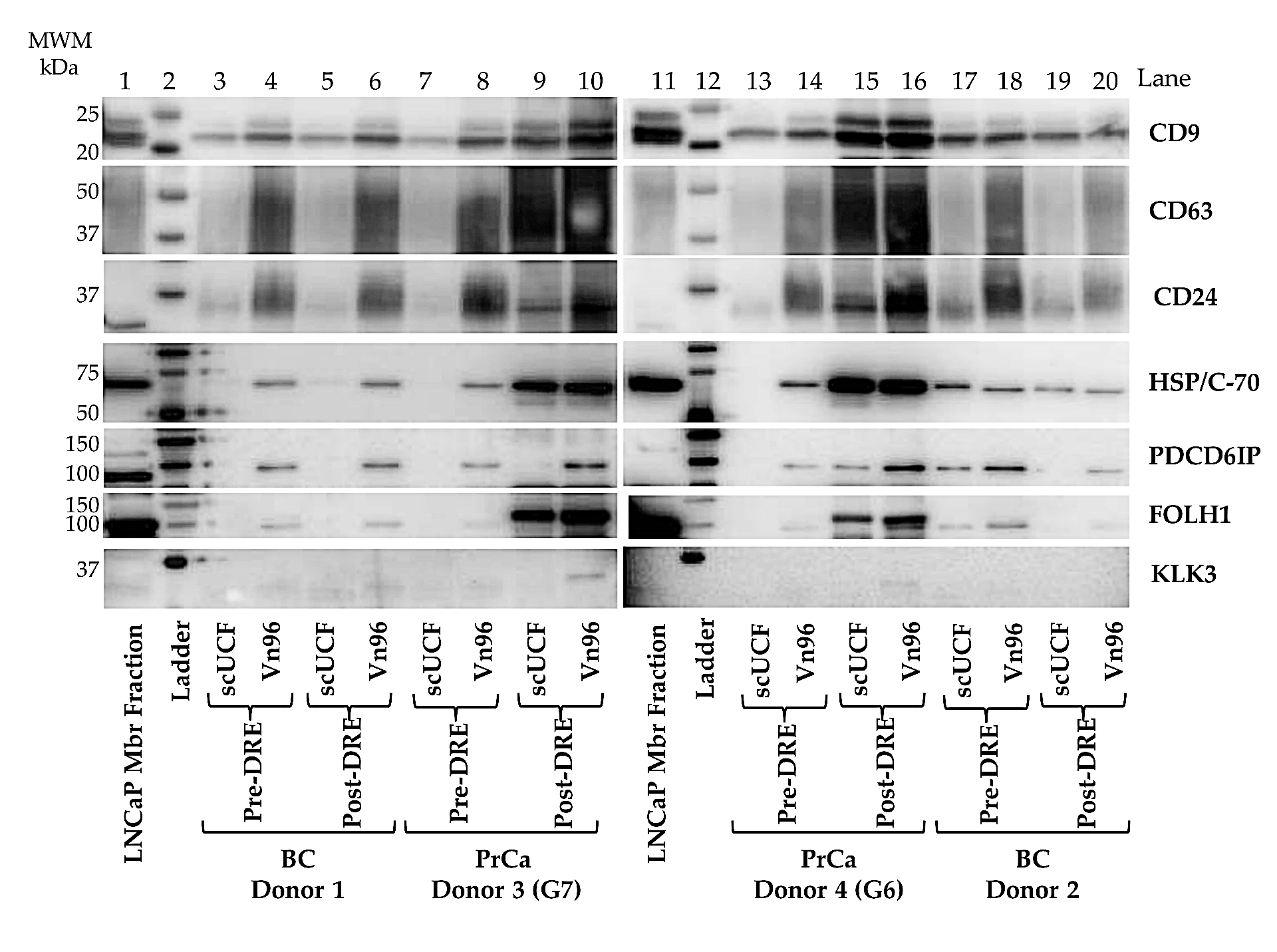

Supplement: Supplementary file 1 [file ijms-21-08330-s001.zip › Figure S1.jpg]

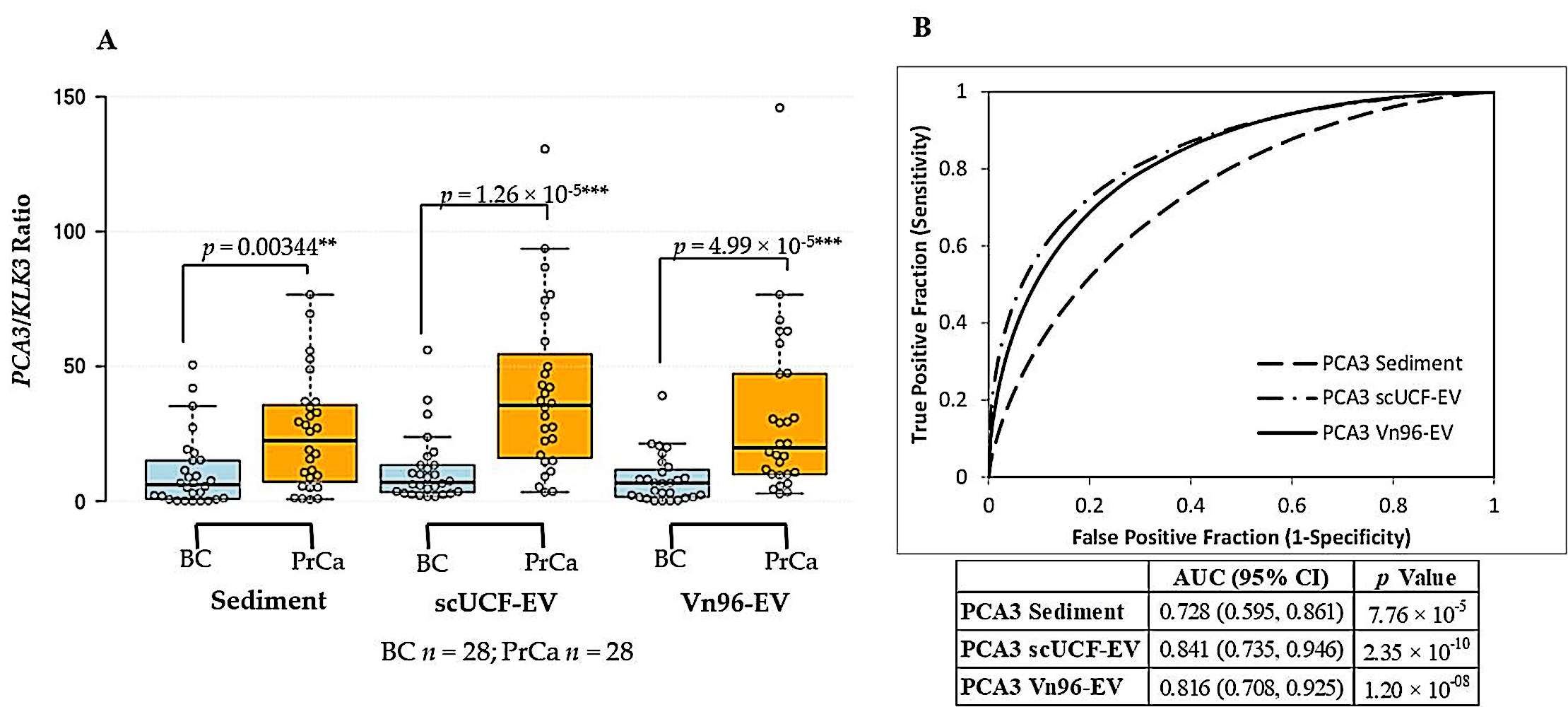

Supplement: Supplementary file 1 [file ijms-21-08330-s001.zip › Figure S2.jpg]
